# Imidazo[1,2-*b*]isoquinoline-5,10-dione

**DOI:** 10.1107/S1600536811022082

**Published:** 2011-06-18

**Authors:** Nassir N. Al-Mohammed, Yatimah Alias, Zanariah Abdullah, Hamid Khaledi

**Affiliations:** a Department of Chemistry, University of Malaya, 50603 Kuala Lumpur, Malaysia

## Abstract

The title butterfly-shaped mol­ecule, C_11_H_6_N_2_O_2_, is folded slightly along the O=C⋯C=O line, the dihedral angle between the two parts being 6.42 (3)°. In the crystal, adjacent mol­ecules are linked through C—H⋯O hydrogen bonds into infinite layers parallel to the *ac* plane. The layers are further connected into a three-dimensional netweork *via* π–π inter­actions formed between pairs of anti­parallel arranged mol­ecules, with a centroid–centroid distance between the central six-membered ring and the benzene ring of 3.4349 (9) Å.

## Related literature

For the structure of isoquinoline­dione-pyrrole fused system in 1,3-dinitro­pyrrolo­[1,2-*b*]isoquinoline-5,10-dione, see: Du & Hitchcock (1992 ▶).
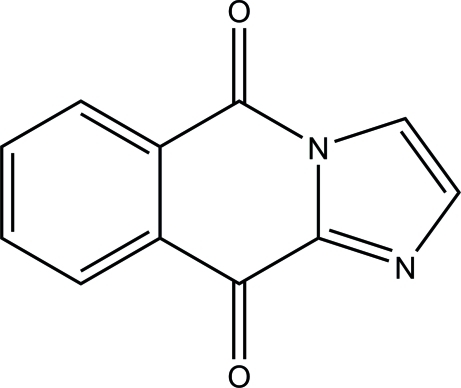


## Experimental

### 

#### Crystal data

C_11_H_6_N_2_O_2_
*M* *_r_* = 198.18
Monoclinic, 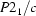

*a* = 7.6518 (7) Å
*b* = 7.2469 (6) Å
*c* = 15.5197 (13) Å
β = 99.947 (1)°
*V* = 847.66 (13) Å^3^
*Z* = 4
Mo *K*α radiation
μ = 0.11 mm^−1^
*T* = 100 K
0.21 × 0.17 × 0.09 mm

#### Data collection

Bruker APEXII CCD diffractometer
Absorption correction: multi-scan (*SADABS*; Sheldrick, 1996 ▶) *T*_min_ = 0.977, *T*_max_ = 0.990
4894 measured reflections
1925 independent reflections
1583 reflections with *I* > 2σ(*I*)
*R*_int_ = 0.021

#### Refinement

*R*[*F* ^2^ > 2σ(*F* ^2^)] = 0.040
*wR*(*F* ^2^) = 0.107
*S* = 1.04
1925 reflections
136 parameters
H-atom parameters constrained
Δρ_max_ = 0.29 e Å^−3^
Δρ_min_ = −0.22 e Å^−3^

### 

Data collection: *APEX2* (Bruker, 2007 ▶); cell refinement: *SAINT* (Bruker, 2007 ▶); data reduction: *SAINT*; program(s) used to solve structure: *SHELXS97* (Sheldrick, 2008 ▶); program(s) used to refine structure: *SHELXL97* (Sheldrick, 2008 ▶); molecular graphics: *X-SEED* (Barbour, 2001 ▶); software used to prepare material for publication: *SHELXL97* and *publCIF* (Westrip, 2010 ▶).

## Supplementary Material

Crystal structure: contains datablock(s) I, New_Global_Publ_Block. DOI: 10.1107/S1600536811022082/gk2380sup1.cif

Structure factors: contains datablock(s) I. DOI: 10.1107/S1600536811022082/gk2380Isup2.hkl

Additional supplementary materials:  crystallographic information; 3D view; checkCIF report

## Figures and Tables

**Table 1:** Hydrogen-bond geometry (Å, °)

*D*—H⋯*A*	*D*—H	H⋯*A*	*D*⋯*A*	*D*—H⋯*A*
C2—H2⋯O1^i^	0.95	2.46	3.2828 (17)	146
C6—H6⋯O1^ii^	0.95	2.57	3.5190 (19)	179
C8—H8⋯O2^iii^	0.95	2.55	3.2233 (17)	128

Symmetry codes: (i) 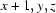; (ii) 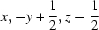; (iii) 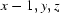.

**Fig. 1.**
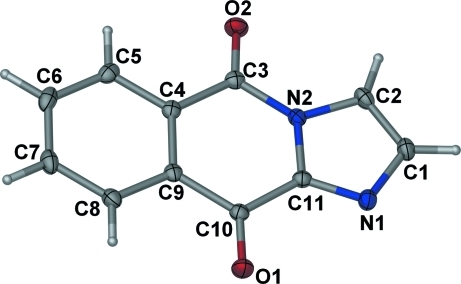
The molecular structure of the title compound showing displacement ellipsoids at the 50% probability level. Hydrogen atoms are drawn as spheres of arbitrary radius.

**Fig. 2.**
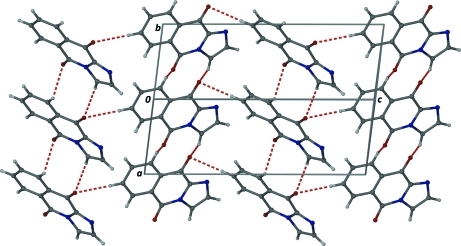
The 2D-network formed by C—H···O hydrogen bonds.

## supplementary crystallographic information

### Comment

This work reports the first crystal structure of an isoquinolinedione-fused imidazole. The structure of an isoquinolinedione-pyrrole fused system has been reported previously (Du & Hitchcock, 1992). The present molecule (Fig. 1) is almost planar (r.m.s deviation = 0.0718 Å) but slighly bent along the O=C···.C=O line with the corresponding dihedral angle of 6.42 (3)°. In the crystal, intermolecular C—H···O interactions (Table 1) connect the molecules into a two-dimensional array in the *ac* plane (Fig. 2). The isoquinoline rings of pairs of the molecule related by symmetry –*x* + 1, -*y* + 1, -*z*, are placed above each other in an anti-parallel manner with the centriods of the six-membered heterocyclic ring and the benzene ring at a distance of 3.4349 (9) Å.

### Experimental

A solution of phthaloyl chloride (5.58 g, 27.5 mmol) in dry pyridine (20 ml) was added dropwise to a mixture of imidazole (1.7 g, 25 mmol) and bis (triphenylphosphine)palladium (II) chloride (0.87 g) in dry pyridine (15 ml) at 273 K. The mixture was refluxed for 4 h, then cooled to room temperature and poured into ice water (150 ml). The aqueous solution was extracted with chloroform and the chloroform solution was washed with 2 % aqueous HCl solution and distilled water (3 x15 ml). It was dried over magnesium sulfate and evaporated under vacuum. The amorphous residue was re-crystallized from acetonitrile to give yellow crystals of the title compound (m.p. = 232-234° C).

### Refinement

Hydrogen atoms were placed at calculated positions and refined as riding atoms with C—H distance of 0.95 Å and with *U*_iso_(H) set to 1.2 *U*_eq_(C).

### Crystal data

**Table:**

C_11_H_6_N_2_O_2_	*F*(000) = 408
*M**_r_* = 198.18	*D*_x_ = 1.553 Mg m^−^^3^
Monoclinic, *P*2_1_/*c*	Mo *K*α radiation, λ = 0.71073 Å
Hall symbol: -P 2ybc	Cell parameters from 1521 reflections
*a* = 7.6518 (7) Å	θ = 2.7–29.5°
*b* = 7.2469 (6) Å	µ = 0.11 mm^−^^1^
*c* = 15.5197 (13) Å	*T* = 100 K
β = 99.947 (1)°	Block, yellow
*V* = 847.66 (13) Å^3^	0.21 × 0.17 × 0.09 mm
*Z* = 4	

### Data collection

**Table:**

Bruker APEXII CCD diffractometer	1925 independent reflections
Radiation source: fine-focus sealed tube	1583 reflections with *I* > 2σ(*I*)
graphite	*R*_int_ = 0.021
φ and ω scans	θ_max_ = 27.5°, θ_min_ = 2.7°
Absorption correction: multi-scan (*SADABS*; Sheldrick, 1996)	*h* = −9→9
*T*_min_ = 0.977, *T*_max_ = 0.990	*k* = −9→8
4894 measured reflections	*l* = −20→19

### Refinement

**Table:**

Refinement on *F*^2^	Primary atom site location: structure-invariant direct methods
Least-squares matrix: full	Secondary atom site location: difference Fourier map
*R*[*F*^2^ > 2σ(*F*^2^)] = 0.040	Hydrogen site location: inferred from neighbouring sites
*wR*(*F*^2^) = 0.107	H-atom parameters constrained
*S* = 1.04	*w* = 1/[σ^2^(*F*_o_^2^) + (0.0535*P*)^2^ + 0.3219*P*] where *P* = (*F*_o_^2^ + 2*F*_c_^2^)/3
1925 reflections	(Δ/σ)_max_ < 0.001
136 parameters	Δρ_max_ = 0.29 e Å^−^^3^
0 restraints	Δρ_min_ = −0.22 e Å^−^^3^

### Special details

**Table:**

Geometry. All e.s.d.'s (except the e.s.d. in the dihedral angle between two l.s. planes) are estimated using the full covariance matrix. The cell e.s.d.'s are taken into account individually in the estimation of e.s.d.'s in distances, angles and torsion angles; correlations between e.s.d.'s in cell parameters are only used when they are defined by crystal symmetry. An approximate (isotropic) treatment of cell e.s.d.'s is used for estimating e.s.d.'s involving l.s. planes.
Refinement. Refinement of *F*^2^ against ALL reflections. The weighted *R*-factor *wR* and goodness of fit *S* are based on *F*^2^, conventional *R*-factors *R* are based on *F*, with *F* set to zero for negative *F*^2^. The threshold expression of *F*^2^ > σ(*F*^2^) is used only for calculating *R*-factors(gt) *etc*. and is not relevant to the choice of reflections for refinement. *R*-factors based on *F*^2^ are statistically about twice as large as those based on *F*, and *R*- factors based on ALL data will be even larger.

### Fractional atomic coordinates and isotropic or equivalent isotropic displacement parameters (Å^2^)

**Table:**

	*x*	*y*	*z*	*U*_iso_*/*U*_eq_	
O1	0.26338 (13)	0.48928 (15)	0.19588 (7)	0.0219 (3)	
O2	0.82600 (13)	0.21685 (15)	0.07219 (7)	0.0224 (3)	
N1	0.62688 (16)	0.52159 (17)	0.28827 (8)	0.0186 (3)	
N2	0.70971 (15)	0.36883 (16)	0.17748 (7)	0.0152 (3)	
C1	0.80899 (19)	0.5005 (2)	0.30252 (10)	0.0202 (3)	
H1	0.8866	0.5453	0.3526	0.024*	
C2	0.86301 (19)	0.4074 (2)	0.23580 (9)	0.0197 (3)	
H2	0.9811	0.3757	0.2304	0.024*	
C3	0.69589 (18)	0.28089 (19)	0.09539 (9)	0.0160 (3)	
C4	0.51463 (18)	0.27899 (19)	0.04292 (9)	0.0154 (3)	
C5	0.4917 (2)	0.20479 (19)	−0.04108 (9)	0.0190 (3)	
H5	0.5907	0.1567	−0.0632	0.023*	
C6	0.3242 (2)	0.2012 (2)	−0.09248 (10)	0.0222 (3)	
H6	0.3089	0.1516	−0.1500	0.027*	
C7	0.1790 (2)	0.2701 (2)	−0.06005 (9)	0.0217 (3)	
H7	0.0644	0.2665	−0.0953	0.026*	
C8	0.20076 (18)	0.3441 (2)	0.02357 (9)	0.0186 (3)	
H8	0.1011	0.3913	0.0454	0.022*	
C9	0.36831 (18)	0.34932 (19)	0.07565 (9)	0.0150 (3)	
C10	0.38834 (18)	0.43063 (19)	0.16455 (9)	0.0155 (3)	
C11	0.57142 (18)	0.44126 (19)	0.21262 (9)	0.0151 (3)	

### Atomic displacement parameters (Å^2^)

**Table:**

	*U*^11^	*U*^22^	*U*^33^	*U*^12^	*U*^13^	*U*^23^
O1	0.0171 (5)	0.0277 (6)	0.0215 (5)	0.0024 (4)	0.0055 (4)	−0.0027 (4)
O2	0.0182 (5)	0.0241 (6)	0.0262 (6)	0.0027 (4)	0.0073 (4)	−0.0027 (5)
N1	0.0194 (6)	0.0195 (6)	0.0161 (6)	−0.0011 (5)	0.0012 (5)	−0.0007 (5)
N2	0.0137 (6)	0.0157 (6)	0.0160 (6)	−0.0003 (4)	0.0022 (4)	0.0007 (5)
C1	0.0191 (7)	0.0209 (7)	0.0189 (7)	−0.0032 (6)	−0.0013 (5)	−0.0004 (6)
C2	0.0145 (7)	0.0201 (7)	0.0229 (7)	−0.0012 (6)	−0.0006 (5)	0.0029 (6)
C3	0.0179 (7)	0.0131 (6)	0.0175 (7)	−0.0002 (5)	0.0044 (5)	0.0017 (5)
C4	0.0185 (7)	0.0120 (6)	0.0156 (7)	−0.0010 (5)	0.0027 (5)	0.0027 (5)
C5	0.0265 (8)	0.0150 (7)	0.0165 (7)	−0.0012 (6)	0.0064 (6)	0.0012 (6)
C6	0.0343 (9)	0.0166 (7)	0.0147 (7)	−0.0056 (6)	0.0015 (6)	0.0003 (5)
C7	0.0226 (7)	0.0215 (8)	0.0186 (7)	−0.0052 (6)	−0.0037 (6)	0.0032 (6)
C8	0.0163 (7)	0.0195 (7)	0.0194 (7)	−0.0017 (6)	0.0017 (5)	0.0029 (6)
C9	0.0169 (7)	0.0134 (7)	0.0146 (7)	−0.0018 (5)	0.0024 (5)	0.0032 (5)
C10	0.0159 (7)	0.0146 (7)	0.0163 (7)	−0.0007 (5)	0.0038 (5)	0.0021 (5)
C11	0.0155 (7)	0.0143 (7)	0.0160 (7)	0.0003 (5)	0.0043 (5)	0.0019 (5)

### Geometric parameters (Å, °)

**Table:**

O1—C10	1.2215 (16)	C4—C9	1.4027 (19)
O2—C3	1.2084 (17)	C5—C6	1.388 (2)
N1—C11	1.3135 (18)	C5—H5	0.9500
N1—C1	1.3812 (19)	C6—C7	1.390 (2)
N2—C11	1.3754 (17)	C6—H6	0.9500
N2—C2	1.3808 (18)	C7—C8	1.387 (2)
N2—C3	1.4121 (18)	C7—H7	0.9500
C1—C2	1.359 (2)	C8—C9	1.3930 (19)
C1—H1	0.9500	C8—H8	0.9500
C2—H2	0.9500	C9—C10	1.4836 (19)
C3—C4	1.4821 (19)	C10—C11	1.4710 (18)
C4—C5	1.3930 (19)		
			
C11—N1—C1	104.80 (12)	C5—C6—C7	120.15 (14)
C11—N2—C2	106.70 (12)	C5—C6—H6	119.9
C11—N2—C3	125.95 (12)	C7—C6—H6	119.9
C2—N2—C3	127.27 (12)	C8—C7—C6	120.21 (14)
C2—C1—N1	111.36 (13)	C8—C7—H7	119.9
C2—C1—H1	124.3	C6—C7—H7	119.9
N1—C1—H1	124.3	C7—C8—C9	120.21 (13)
C1—C2—N2	105.33 (13)	C7—C8—H8	119.9
C1—C2—H2	127.3	C9—C8—H8	119.9
N2—C2—H2	127.3	C8—C9—C4	119.48 (13)
O2—C3—N2	120.33 (13)	C8—C9—C10	119.16 (12)
O2—C3—C4	125.01 (13)	C4—C9—C10	121.35 (12)
N2—C3—C4	114.65 (12)	O1—C10—C11	121.49 (13)
C5—C4—C9	120.00 (13)	O1—C10—C9	123.10 (13)
C5—C4—C3	118.17 (12)	C11—C10—C9	115.39 (12)
C9—C4—C3	121.83 (12)	N1—C11—N2	111.81 (12)
C6—C5—C4	119.95 (13)	N1—C11—C10	127.51 (12)
C6—C5—H5	120.0	N2—C11—C10	120.63 (12)
C4—C5—H5	120.0		

### Hydrogen-bond geometry (Å, °)

**Table:**

*D*—H···*A*	*D*—H	H···*A*	*D*···*A*	*D*—H···*A*
C2—H2···O1^i^	0.95	2.46	3.2828 (17)	146
C6—H6···O1^ii^	0.95	2.57	3.5190 (19)	179
C8—H8···O2^iii^	0.95	2.55	3.2233 (17)	128

Symmetry codes: (i) *x*+1, *y*, *z*; (ii) *x*, −*y*+1/2, *z*−1/2; (iii) *x*−1, *y*, *z*.
